# Synthetic rewiring and boosting type I interferon responses for visualization and counteracting viral infections

**DOI:** 10.1093/nar/gkaa961

**Published:** 2020-11-02

**Authors:** Natascha Gödecke, Jan Riedel, Sabrina Herrmann, Sara Behme, Ulfert Rand, Tobias Kubsch, Luka Cicin-Sain, Hansjörg Hauser, Mario Köster, Dagmar Wirth

**Affiliations:** RG Model Systems for Infection and Immunity, Helmholtz Centre for Infection Research, Braunschweig 38124, Germany; RG Model Systems for Infection and Immunity, Helmholtz Centre for Infection Research, Braunschweig 38124, Germany; RG Model Systems for Infection and Immunity, Helmholtz Centre for Infection Research, Braunschweig 38124, Germany; RG Model Systems for Infection and Immunity, Helmholtz Centre for Infection Research, Braunschweig 38124, Germany; Department of Vaccinology and Applied Microbiology, Braunschweig 38124, Germany; Department of Vaccinology and Applied Microbiology, Braunschweig 38124, Germany; Department of Vaccinology and Applied Microbiology, Braunschweig 38124, Germany; Centre for Individualised Infection Medicine (CiiM), a joint venture of Helmholtz Centre for Infection Research and Hannover Medical School, Hannover 30625, Germany; Staff Unit Scientific Strategy, Helmholtz Centre for Infection Research, Braunschweig 38124, Germany; RG Model Systems for Infection and Immunity, Helmholtz Centre for Infection Research, Braunschweig 38124, Germany; RG Model Systems for Infection and Immunity, Helmholtz Centre for Infection Research, Braunschweig 38124, Germany; Institute of Experimental Hematology, Medical University Hannover, Hannover 30625, Germany

## Abstract

Mammalian first line of defense against viruses is accomplished by the interferon (IFN) system. Viruses have evolved numerous mechanisms to reduce the IFN action allowing them to invade the host and/or to establish latency. We generated an IFN responsive intracellular hub by integrating the synthetic transactivator tTA into the chromosomal Mx2 locus for IFN-based activation of tTA dependent expression modules. The additional implementation of a synthetic amplifier module with positive feedback even allowed for monitoring and reacting to infections of viruses that can antagonize the IFN system. Low and transient IFN amounts are sufficient to trigger these amplifier cells. This gives rise to higher and sustained—but optionally de-activatable—expression even when the initial stimulus has faded out. Amplification of the IFN response induced by IFN suppressing viruses is sufficient to protect cells from infection. Together, this interfaced sensor/actuator system provides a toolbox for robust sensing and counteracting viral infections.

## INTRODUCTION

Viruses cause a plethora of various diseases and interfere with the host's regulatory networks in a versatile manner to facilitate their dissemination or latency establishment. To overcome viral infections, mammals have evolved innate as well as adaptive immune responses. The type I IFN system is part of the highly conserved innate immune response and represents the first line of defence against invading viruses (1) but is also induced upon bacterial infection (2). Pattern recognition receptors specifically detect components of incoming pathogens, so-called pathogen associated molecular patterns (PAMPs) and lead to the secretion of early type I IFNs. Type I IFNs are sensed by infected cells as well as by non-infected cells via the cognate IFN-α/β receptor (IFNAR) which is expressed on virtually all cell types. Binding to the receptor is followed by activation of receptor-associated tyrosine kinases of the JAK family, which in turn phosphorylate the transcription factors STAT1 and STAT2 (3). Upon heterodimerization, STAT1/2 complexes bind to interferon regulatory factor 9 (IRF9), forming the trimeric transcription factor ISGF3. ISGF3 can translocate into the nucleus and bind to a panel of promoters characterized by an IFN-stimulated response element IFN specific regulation element (ISRE), finally culminating in the transcriptional activation of several hundred genes (IFN-stimulated genes, ISGs). Together, this concerted activation of cellular genes results in the establishment of a highly protective antiviral state which inhibits key steps of the viral life cycle (4).

To avoid unintended pathological consequences associated with prolonged or overshooting IFN responses, the activation of the IFN system needs to be tightly controlled. Various negative feedback mechanisms can limit the activation of the IFN production (5) and the activation of ISGs. Control elements of the IFN response include the internalisation of the receptor as well as the induction of negative regulators (ubiquitin carboxy-terminal hydrolase 18 (USP18), suppressors of cytokine signaling (SOCS)), and regulatory miRNAs (6). As a consequence, the cellular IFN response is transient, which is sufficient to protect the host during the first days of infection, before adaptive immune responses build up the long-term, sustained immunity.

The multistep regulation of the IFN response provides also multiple targets for viral antagonistic mechanisms. Accordingly, during evolution, viruses have developed a broad portfolio of potent counteracting strategies that terminate the IFN response early after infection and thus facilitate viral propagation (7).

For monitoring the activation of the IFN system and the induced antiviral measures, sensitive reporter systems have been generated by substituting the chromosomal IFN-β gene for visualizing genes such as luciferase (8–10). Likewise, reporter systems reflecting the activation of the IFN response pathway have been created. The antiviral Myxovirus resistance gene 2 (Mx2), a prototypical ISG, was used to monitor activation of IFN signaling upon sensing by the IFN-α/β receptor in both, cells and mice (11,12). Of note, Mx2 expression correlates well with the antiviral state of IFN-stimulated cells, thus qualifying Mx2-based systems as authentic reporters (13). Generally, these IFN-based reporter systems offer the opportunity to monitor infections with viruses (13) as well as with bacteria (14) with spatial and temporal resolution. Reflecting the activation of the underlying intrinsic signaling pathways, both IFN induction reporters and IFN response reporters are characterized by a restricted time of activation.

The application of cells that can sense and indicate a disease state and produce an appropriate therapeutic response represents a highly attractive option, albeit not yet implemented in medicine (15). Such ‘theranostic’ cells can be constructed based on tailor-made synthetic sensor/actuator modules which take up physiological signals and rewire them to synthetic expression cassettes. Synthetic biology provides tools to equip cells with regulatory modules, thereby facilitating controlled activation (16). A paradigm synthetic regulatory module for mammalian cells represents the Tet-off system (17). This system is based on a synthetic transactivator (tTA) which binds the synthetic P_Tet_ promoter and activates transcription of downstream genes. Upon addition of doxycycline, binding is prevented, thereby abrogating gene induction (tet-off). Synthetic P_Tet_ promoters realize tight as well as time and dose-dependent transgene expression, controlled by external addition of doxycycline. Moreover, various synthetic expression modules can be combined to complex cascades, e.g. for signal amplification or for establishment of Boolean networks (18). In emerging synthetic biology concepts, these building blocks are interfaced with natural cellular regulatory networks (reviewed in (19,20)). Rewiring physiological triggers to synthetic switches provides the option to create autonomously controlled cascades. Moreover, such networks can be fine-tuned by employing additional external cues for controlling the synthetic cassettes. Such interfaced sensor/actuator systems provide the option to visualize and/or even overcome particular disease states *in vitro* and—upon transplantation—also *in vivo* (15–16,21–22).

In the current study, we aimed at exploiting the type I IFN system as an immediate and highly sensitive infection detector to initiate potent and sustained antiviral countermeasures, thereby facilitating even the combat of viruses that can counteract the IFN response. To this end, we utilized the IFN response pathway and created an authentic converter module to redirect ISG activation towards synthetic P_Tet_ promoter induction. We show that this infection-controlled hub can be connected to genes of choice. Such converter modules generate a controlled and transient program mimicking the antiviral state and at the same time allow external control of the actuator component via doxycycline administration. By addition of a P_Tet_ amplifier module we generated cells with an all-or-nothing sensitivity and sustained expression even when the trigger is removed. Moreover, we rewired the IFN response pathway to induce an antiviral program that protects other cells in the population from infection. Since these modules can be integrated in diverse mammalian cells they represent a prototype for the development of highly sensitive sensor-actuator systems to efficiently fight infections.

## MATERIALS AND METHODS

### Cells and culture conditions

All cells were maintained in DMEM (GIBCO, Carlsbad, CA, USA) supplemented with 10% fetal calf serum, 2 mM l-glutamine, non-essential amino acids, HEPES (10 mM), penicillin (10 U/ml) and streptomycin sulfate (100 μg/ml).

Mx-tTA mouse embryonic stem (ES) cells (see Supplementary Figure S1 for details of the generation) were cultivated at 37°C, 7% CO_2_ and 2% O_2_ at maximal humidity on gelatinized feeder cell-covered tissue flasks in order to maintain pluripotency of the cells. Differentiated Mx-tTA cells were cultivated at 37°C in a humidified atmosphere with 5% CO_2_. For generating differentiated Mx-tTA cell line, the CRISPR Cas modified ES Mx-tTA cells were seeded on 6-well plates and cultivated in absence of LIF. When cells became confluent, they were passaged in a 1:4 ratio. After 10 days of cultivation, the fibroblastoid cells were infected with a recombinant lentivirus transducing SV40 T antigen according to a previously published protocol (23). If not indicated differently, the experiments were performed with the differentiated and immortalized Mx-tTA cell line.

mIFN-β was produced from recombinant BHK-21 cells as described earlier (12). mIFN-β activity was measured using a bioassay based on Mx2-Luc reporter gene expression (11).

### Cell engineering

Transfection was performed using Lipofectamine 2000 (Invitrogen) according to the manufacturer's protocol. In brief, one day prior transfection cells were seeded to reach 70–80% confluency the next day. On the day of transfections, cells were washed with PBS and incubated with a mixture of 4 μg plasmid DNA and Lipofectamine 2000 in serum-free media for 6–12 h and subsequently cultured in standard FCS containing media. One day after transfection the respective selection drug was added and resistant cell pools with stably integrated cassettes were selected. For selection, the following drugs were used: Puromycin dihydrochloride (2.5 ug/ml, Gibco), Blasticidin (4 ug/ml, InvivoGen), Hygromycin (Calbiochem, 300 U/ml) and G418 (0.8 mg/ml, Fisher Scientific).

The Conv cells were constructed by stable transfection of a pRBT1Luc (24) in which firefly luciferase is under the control of a bidirectional P_Tet_ promoter. To facilitate selection, a hygromycin phosphatase expression cassette under the control of a constitutive promoter was co-transfected.

The ConvAmp cells were generated from Conv cells by transfection of pRBTTtTA (25) which harbours a P_Tet_-tTA module. To select for stable integration into the cellular genome, a puromycin expression cassette under the control of a constitutive promoter was co-transfected.

The ConvAmp-eGFP cells were constructed by stable transfection of ConvAmp cells with a plasmid encoding a P_Tet_-eGFP module as well as an P_mPGK_ driven puromycin acetyl transferase gene.

The Conv-hIFN cells were generated from Conv cells by transfection of a plasmid encoding a P_Tet_-IFN module as well as a constitutive P_mPGK_ driven puromycin acetyl transferase gene.

### Viral infections

Infections with Newcastle disease virus (NDV) La Sota strain (Lohmann Tierzucht, Cuxhaven, Germany) and Vesicular Stomatitis virus containing an eGFP reporter (26) were performed in serum-free media using MOIs as indicated in the legends. 1 h after infection, residual virus was removed and cells were washed and cultured in serum-containing medium.

For tracking mCMV infection, MCMV mCherry-P2A-ie1/3 was generated by *en passant* BAC mutagenesis (27) on a MCMV Smith strain background (GenBank: GU305914.1) using the BAC pSM3fr-MCK-2fl (clone 3.3) (28). The viral gene encoding immediate early transcripts 1 and 3 (m122/123)) was fused with the gene encoding red fluorescent protein mCherry. The P2A peptide-encoding sequence was inserted after mCherry removing its stop codon to maintain the ORF. All cloning design was carried out on SnapGene software (GSL Biotech, USA). The recombinant BAC was transfected into NIH3T3 cells using FuGene HD (Promega, USA) and reconstituted viral particles were passaged five times before generating a stock from infected Balb/c MEF cells. MCMV infection was performed after washing the cells with PBS followed by 1 h incubation in serum free media.

### RT-qPCR

Total RNA was prepared from ∼1 × 10^5^ cells using the RNeasy Mini Kit (Qiagen, Hilden, Germany) following the manufacturer's instructions. The RNA samples were eluted in 30–50 μl RNase-free H_2_O. Quality and quantity of the isolated RNA samples were assessed using the NanoDrop ND-100 spectrophotometer. Reverse transcription was carried out using the Ready-To-Go First Strand Beads (GE Healthcare) and OligodT primers (Qiagen) according to the manufacturer's instructions. Briefly, a volume corresponding to 5 μg of eluted RNA was denatured at 65°C for 10 min, followed by 5 min incubation on ice and then mixed with OligodT primers and transferred to the Ready-To-Go First Strand Beads for reverse transcription at 37°C for 1 h. 1:10 diluted cDNAs were used as a template for amplification with specific primers for coding sequences of tTA (tTA1: 5′-GGACGAGCTCCACTTAGACG, tTA2 5′AGGGCATCGGTAAACATCTG-) and Mx2 (Mx2fwd: 5′-TCACCAGAGTGCAAGTGAGG;Mx2rev: 5′-CATTCTCCCTCTGCCACATT).

For real-time PCR the following components were mixed: 10μl SYBR green (Qiagen) RT-PCR mix, 1 μl forward primer (10 pmol), 1 μl reverse primer (10 pmol), 8 μl cDNA mix. Real-time PCR was performed on a LightCycler 480 apparatus (Roche). The housekeeping gene actin (Actinfwd: 5′-TGGAATCCTGTGGCATCCATGAAAC Actinrev: 5′-TAAAACGCAGCTCAGTAACAGTCCG) was used for normalization. Reactions were performed in duplicates or triplicates.

### Flow cytometry analysis

For flow cytometry, cells were detached from adherent culture by enzymatic treatment and resuspended in a buffer (2% FBS in PBS). Flow cytometry analysis was performed on an LSR-II SORP and FACS-Calibur (BD Biosciences). Data were processed using FlowJo V10.

### *In vitro* luciferase measurement

The cells were washed in PBS and harvested in 250 μl RLB Buffer (Promega, E397A). 20 μl of this solution was mixed with 100 μl detection solution (Beetle Lysis Juice, PJK) and measured using the luminometer Lumat LB 9507 (Berthold). The protein content was measured with Nanodrop biophotometer (Eppendorff). Relative light units (RLU) obtained from 10 s were related to the protein content (RLU/mg).

### Imaging

For live cell imaging, cells were seeded into ibidi μ-slides. Images were acquired on a Zeiss LSM 980 confocal microscope using a 20×/0.8 Plan Apochromat objective or a 10×/0.45 Plan Apochromat objective in an imaging chamber maintained at 37°C with 5% CO_2_. Microscopical picture series were analysed with ImageJ (NIH, Bethesda, MD, USA).

### Statistical evaluation

All data analyses were performed using GraphPad Prism 8.3.0. If not stated differently, the figures represent mean values and standard deviations based on the sample size as specified for the particular experiments.

## RESULTS

### Conversion of type I IFN towards luciferase expression

**Figure 1.**
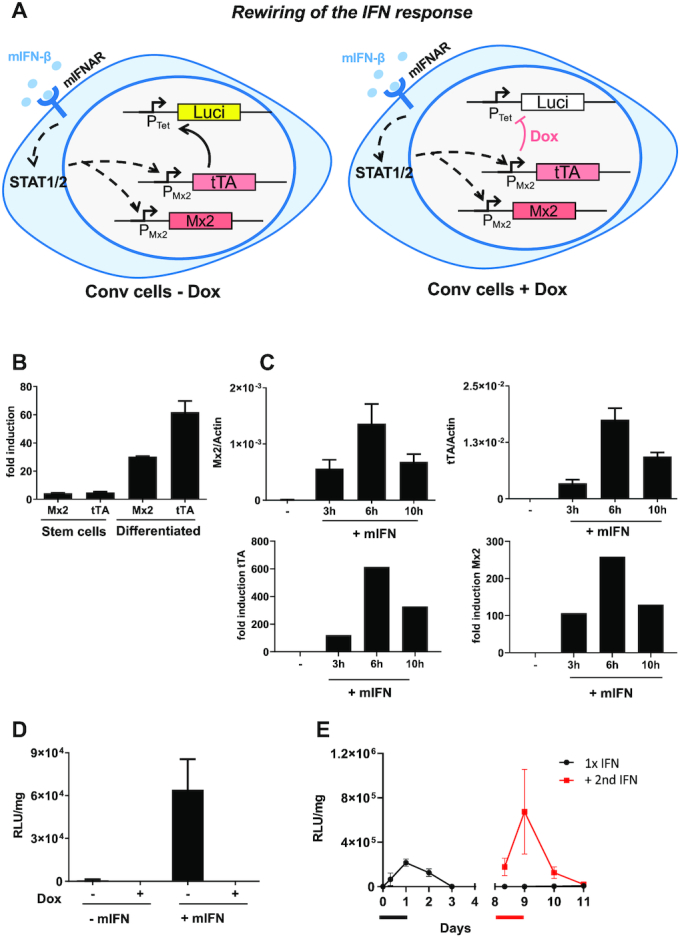
mIFN-β and doxycycline-controlled expression in Conv cells. (**A**) Depiction of the intracellular circuit in Conv cells for rewiring and switching-off of expression in presence of doxycycline (Dox). The transcriptional activator tTA is integrated downstream of the Mx2 promoter (Mx-tTA cells, see Supplementary Figure S1 for details) and expressed only upon stimulation of IFNAR with mIFN. tTA binds to its cognate P_Tet_ promoter and activates luciferase expression. Doxycycline blocks binding of tTA and thereby switches off expression. Coloured boxes indicate induced genes upon IFN stimulation, white boxes indicate the situation in the repressed state (+Dox). (**B**) Relative Mx2 and tTA mRNA levels were determined in Mx-tTA embryonic stem cells and in *in vitro*-differentiated primary cells derived thereof. Upon stimulation of cells with 500 U/ml mIFN-β for 24h, mRNA was isolated. Mx2, tTA and actin levels were quantified by RT-qPCR. The fold induction of induced vs. non-induced cells is depicted. The presented data were derived from a single experiment done in triplicates. Mean values (**C**) mRNA levels of Mx2 and tTA were determined upon stimulating MxtTA cells with 50 U/ml mIFN-β for the indicated time. The relative expression in relation to actin is shown. The presented data are based on five samples derived from two independent experiments. (**D**) Luciferase expression upon stimulating Conv cells with 500 U/ml mIFN-β for 24 h in presence and absence of Doxycycline. Luciferase was analysed after withdrawal of mIFN. The presented data are based on six samples derived from two independent experiments. (**E**) Kinetics of luciferase expression in Conv cells. Cells were transiently stimulated with 500 U/ml IFN-β for 24 h and then analysed over time (black line). A fraction of cells was restimulated on day 8 with 500 U/ml mIFN-β for 24 h and monitored (red line). The time of first and second mIFN-β induction is represented by the black and red bars. The presented data were derived from a single experiment out of several other experiments with comparable settings and was performed in triplicates.

To generate virus responsive sensor/actuator systems we developed type I IFN converter (Conv) cells in which stimulation of the IFN receptor (IFNAR) and activation of the cellular Jak/STAT signaling pathway leads to the expression of the doxycycline regulatable transcriptional transactivator tTA (Figure 1A). Mx-tTA embryonic stem (ES) cells were generated by targeting the tTA gene into the endogenous Mx2 locus by CRISPR/Cas9-assisted homologous recombination, thereby replacing one of the two alleles of the cellular Mx2 gene (for details see Supplementary Figure S1). The mRNA expression levels of tTA and Mx2 were determined both in genetically engineered ES cells as well as in fibroblastoid cells derived thereof by in vitro differentiation. Upon 24 hours of mouse IFN-β (mIFN-β) treatment, the tTA and the Mx2 transcript abundance was increased in both cell types. mRNA levels were induced about 5-fold for both genes in the ES cells, while in the differentiated cells, 40-fold and 60-fold higher levels were found for Mx2 and tTA, respectively (Figure 1B). The higher activation levels in the differentiated cells are in agreement with previous reports showing that in stem cells high expression of the cellular counter regulator SOCS1 attenuates full blown induction of ISGs (29). IFN-induced Mx2 and tTA mRNA expression levels were monitored in immortalized Mx-tTA cells. Both genes displayed similar expression kinetics with a peak induction at 6 h (258-fold and 613-fold for Mx2 and tTA, respectively) indicating that tTA expression mirrors endogenous Mx2 expression (Figure 1C) as well as other ISGs (Supplementary Figure S2). In addition, the resistance of IFN-treated cells towards infection reflects the induction of a functional antiviral program (Supplementary Figure S3). When redirecting IFNAR activation to a stably transfected P_tet_-driven luciferase cassette in converter (Conv) cells, we observed strong induction of luciferase activity 24 h upon stimulation with mIFN-β. However, when Conv cells additionally received Doxycycline, luciferase expression was completely abrogated, indicating that expression crucially relies on tTA (Figure 1D). Next, the kinetics of luciferase activity was determined in response to a 24 h mIFN trigger. A peak of luciferase expression declined to baseline levels (Figure 1E). A second pulse of mIFN induction led to reporter re-stimulation and subsequent decline to baseline levels. Note that the elevated levels of luciferase activity after restimulation is a result of ISG-mediated memory effect (priming) (13,30–31).

### Sensing of viral infections by the Conv module

**Figure 2.**
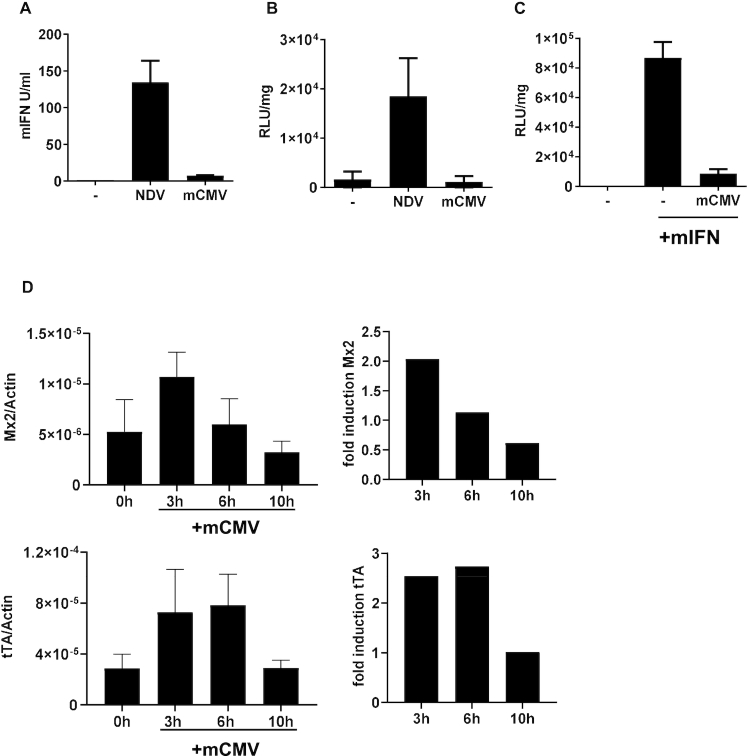
Conv cells respond to NDV but not to mCMV infection. (**A**) The IFN levels were determined in the supernatants of Conv cells upon infection with NDV (80 HA units/ml) and mCMV (MOI 6), respectively, on day 2 upon infection. The presented data are based on six (NDV) or three (mCMV) samples from two and one independent experiments, respectively. (**B**) Conv cells were infected with NDV (80 HA units/ml) and mCMV (MOI 6) for one hour. Virus was removed and luciferase expression of Conv cells was determined 24 h afterwards. The presented data are based on six (NDV) or three (mCMV) samples of two independent experiments. (**C**) mCMV-mediated blocking of the IFN response pathway was determined by infecting Conv cells with mCMV (MOI 6) for 1 h and subsequently stimulating with 50 U/ml mIFN-β for 24 h. Luciferase expression was analysed 24 h after infection. The experiment was performed twice in triplicates. (**D**) Conv cells were infected with mCMV (MOI 6) for 1 h. The relative expression of endogenous Mx2 and synthetic tTA was determined by RT-qPCR in absence of virus as well as at the indicated time points after infection with mCMV. The fold induction was calculated in relation to non-infected cells. The presented data are based on 4–6 samples derived from two independent experiments.

In order to examine if Conv cells can directly sense viral infections via induction of endogenous mIFN the LaSota strain of Newcastle Disease Virus (NDV) was employed. This virus replicates and induces mIFN expression in murine cells but shows no interference with innate immune signaling (9,32). Further, we selected the mouse restricted Cytomegalovirus (mCMV) as an example for a virus that interferes with the IFN system at different levels (33). NDV-infected cells showed a strong increase (100-fold) of mIFN secretion, while the production of type I mIFN was only slightly elevated (6-fold) during mCMV infection (Figure 2A). The induction of luciferase in the infected Conv cells corresponded to the mIFN levels (Figure 2B). This indicates that the NDV-induced cellular mIFN production was sufficient to activate Conv cells, which rewired the endogenous IFNAR signal to the P_Tet_ promoter. In contrast, mCMV-infected Conv cells showed no measurable induction of luciferase activity although flow cytometry confirmed an infection efficiency of ∼80% (Figure 2B and Supplementary Figure S4). The data from Figures 2A and B suggest that the antagonistic action of mCMV reduces production of mIFN and efficiently blocks the IFN response pathway. To verify that mCMV inhibits IFN activity, we first infected Conv cells with mCMV and added exogenous mIFN-β 24 h post-infection. Notably, luciferase induction was widely abrogated in infected cells, suggesting that mCMV infection strongly blocks the IFN response in Conv cells (Figure 2C).

These data are further in line with an inhibition of IFNAR signaling by the interference of the viral m27 protein with cellular STAT2 (34). To identify the extent of residual CMV-mediated ISG induction mRNA levels of the endogenous Mx2 gene as well as those of the tTA gene were determined after mCMV infection. Virus infection rapidly triggered the transcriptional induction of both genes. However, both mRNAs were only induced ∼2-fold (Figure 2D), which is much less than the expression levels obtained by stimulation of the cells with 50 U/ml mIFN-β (cf. Figure 1C). Moreover, Mx2 and tTA mRNA levels reverted to background levels within 6 and 10 h after infection, respectively. The limited level and the transient nature of induction is also reflected by transient expression of other ISGs (Supplementary Figure S5). Together, the results indicate that mCMV infection induces a weak type I IFN activity in Conv cells. Despite viral antagonism with Jak/STAT signaling, residual ISG induction is measurable and is sufficient to trigger the converter module.

### Implementation of a synthetic positive feedback loop facilitates sustained conversion of weak trigger signals into an effective antiviral response

**Figure 3.**
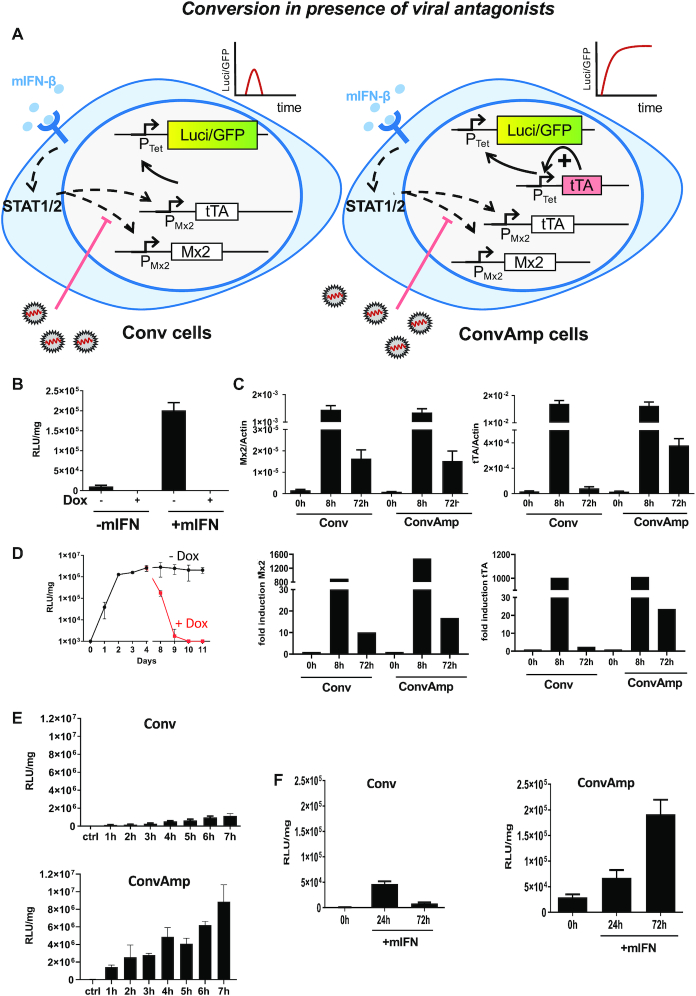
Implementation of a positive feedback module for increased and sustained IFNAR-driven transgene expression. (**A**) Schematic depiction upon transient activation of Conv cells as well as the ConvAmp cells, e.g. in presence of antagonistic viral proteins. Coloured boxes indicate constantly induced genes, white boxes indicate the situation upon viral counteraction. ConvAmp cells were generated upon integrating the P_Tet_-tTA positive feedback module into Conv cells. As actuators, P_Tet_-luc and P_Tet_-EGFP are depicted. (**B**) Luciferase expression of ConvAmp cells was determined 24 h after stimulation with 500 U/ml mIFN-β in the presence and absence of doxycycline. Data are based on one out of two independent experiments performed in triplicates. (**C**) The kinetics of relative Mx2 and tTA mRNA levels were determined by RT-qPCR 8 h or for 72 h after stimulating Conv and ConvAmp cells with mIFN-β (500 U/ml). The experiment was performed twice in triplicates for the untreated and 72 h timepoints and twice in duplicates and triplicates for the 8 h timepoint. (**D**) The time course of luciferase expression was determined in ConvAmp cells upon a transient stimulation with 500 U/ml mIFN-β for 24 h (black line). From day 8, a subpopulation of cells was cultured in presence of doxycycline (red line). The experiment was performed once in triplicates. (**E**) The luciferase expression in Conv and ConvAmp cells was determined after pulsing with 500 U/ml mIFN-β for the indicated time. Samples were harvested 24 h after the start of mIFN-β treatment. The experiment was performed once in triplicates. (**F**) The kinetics of luciferase expression was determined after pulsing Conv and ConvAmp cells with 50 U/ml mIFN-β for 6 h. Luciferase expression was determined at 24 and 72 h. Data are based on one out of two independent experiments performed in triplicates.

The results about the response to mCMV as outlined above indicate that even under strong viral antagonism a residual activation of ISG expression is detectable. We aimed to bypass the viral antagonism in order to gain a potent antiviral response by amplification of the signal. Further, we wanted to convert a transient trigger into a sustainable out-put that is independent of the trigger. Both aims were achieved by equipping Conv cells with a P_Tet_-tTA module (35). The module, once activated, establishes a positive feedback loop in which the P_Tet_ promoter induces expression of the tTA (Figure 3A). Importantly, in these converter/amplifier (ConvAmp) cells the initial activation of the synthetic loop still depends on virus- or IFN-mediated Mx-tTA induction. However, once initiated, the outgoing signal is maintained by autoregulation of the tTA protein. To characterize the ConvAmp cells, we analysed luciferase expression 24 h after mIFN-β stimulation in the presence or absence of doxycycline. The ConvAmp cells revealed a strong induction of luciferase expression in response to mIFN-β, which was completely suppressed by addition of doxycycline (Figure 3B). If compared to Conv cells (see Figure 1D), luciferase activity was much higher, indicating that implementation of the positive feedback module provided higher sensitivity towards induction of the actuator while the mRNA levels of cellular ISGs were comparable between the two cell lines (Supplementary Figure S2). We determined the consequences of a transient mIFN-β trigger on mRNA levels of tTA and endogenous Mx2 in ConvAmp cells as well as in the parental Conv cells. After 8 h of mIFN-β stimulation we observed corresponding induction levels of both mRNAs in both cell lines (Figure 3C). The kinetics of Mx2 mRNA induction to an mIFN-β pulse were comparable and declined to the same level, which was similarly reflected also for other ISGs (Supplementary Figure S6).

Notably, the tTA levels 72 h after stimulation were found to be higher in ConvAmp cells compared to Conv cells (Figure 3C). We therefore conclude that in ConvAmp cells the initial tTA expression levels (after 8 h) mainly depend on mIFN-β-mediated Mx2 promoter induction while the tTA mRNA levels at later time points (after 72 h) become independent of Mx2 promoter activity and are mediated solely by the synthetic positive feedback module.

We assessed if the sustained tTA expression would result in functional conversion of the signal to luciferase over time. Treatment of ConvAmp cells with mIFN-β for 24h revealed sustained luciferase expression for at least 10 days. Of note, luciferase activity increased during the first 2 days and then plateaued until the end of the experiment, indicating that high levels of luciferase expression could be achieved persistently (Figure 3D). This indicates that an mIFN-triggered signal can be converted into sustained transgene activation in ConvAmp cells. As expected, doxycycline administration at day 7 led to a drop of reporter activity to background levels, confirming that at this stage luciferase expression was completely dependent on tTA activity and independent of IFNAR signaling.

For further characterization we applied different pulses of exogenous mIFN-β to activate the positive feedback loop of the P_Tet_-tTA module and checked whether this is sufficient to maintain expression in ConvAmp cells. ConvAmp cells as well as the Conv cells as control were pulsed with a low and high dose of mIFN-β (50 and 500U/ml) for 1–7 h and luciferase activity was determined (Figure 3E and Supplementary Figure S7). ConvAmp cells proved to be more sensitive and showed higher luciferase activity after the pulses with mIFN-β compared to Conv cells for both doses. In ConvAmp cells, stimulation with 500 U/ml for 1 h was sufficient to convert the signal and switch on the synthetic reporter to a higher extent than the luciferase levels of Conv cells after triggering for 7 h. Similarly, stimulation with 50U/ml activated the reporter after 2–3 h in the ConvAmp cells, while it took 5 h to induce reliably luciferase expression in Conv cells.

We tested if the implementation of the amplifier loop in ConvAmp would allow long-term visualization of a weak trigger signal. To this end, ConvAmp cells as well as Conv cells were stimulated with a low dose for 6 h and luciferase activity was measured at 24 and 72 h. These conditions induced reporter gene expression in both cell systems. However, while Conv cells displayed minor and transient upregulation of luciferase activity, in ConvAmp cells luciferase expression was activated at 24 h and further increased at 72 h (Figure 3F). Thus, the positive feedback amplifier module in the ConvAmp cells boosted and sustained expression beyond clearance of the initial mIFN-β trigger. Together, the results show that the amplifier loop effectively increases the sensitivity of the Mx-tTA module and bypasses negative feedback regulation of the native IFN system.

**Figure 4.**
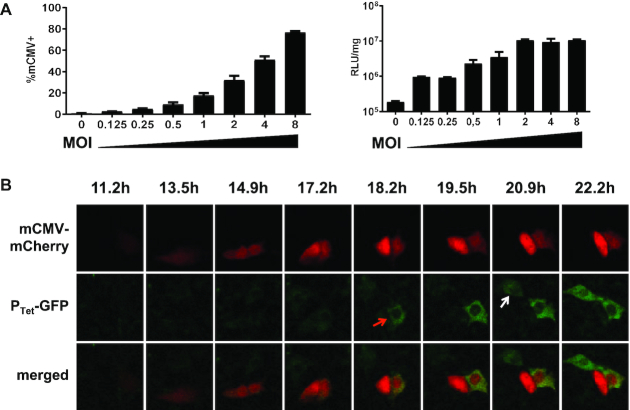
Sensitivity of ConvAmp cells toward mCMV infection. (**A**) The sensitivity of ConvAmp cells in response to mCMV infection was determined. ConvAmp cells were infected with an tRFP-tagged mCMV using indicated MOIs. Frequencies of infected cells were determined by flow cytometry (left, 20 h after infection). Luciferase responses were evaluated 24 h after infection (right). Shown are the mean of duplicates. (**B**) ConvAmp-eGFP cells were seeded one day prior mCMV infection. Cells were infected with mCMV (MOI 1) for 1 h and subjected to time-lapse microscopy. Time points indicate image acquisition post-infection. Representative pictures of one out of two experiments are shown. Luciferase expression of mCMV infected ConvAmp cells is provided in Supplementary Figure S10.

We asked whether the improved properties of ConvAmp cells would allow sensing infections with the strongly antagonizing mCMV. ConvAmp cells were infected with an mCherry-tagged mCMV reporter virus at different MOIs. Using flow cytometry, the percentage of infected cells were determined after infection with increasing viral titres. 80% mCherry-positive cells were detected at an MOI of 8 (Figure 4A). Luciferase activity was determined 24 h post-infection. Even MOIs as low as 0.1 increased the reporter gene expression 5-fold (Figure 4A). Notably, infections with MOIs of 2 and higher induced maximal activity of the reporter in ConvAmp cells, indicating high sensitivity towards mCMV which is not seen in Conv cells (cf. Figure 2A). These results demonstrate the pronounced sensitivity of the ConvAmp system even in conditions when the number of infected cells is hardly detectable (MOI of 0.125, Figure 4A) and also for viruses that strongly interfere with the antiviral IFN signaling.

IFN can induce antiviral responses in both, the infected cells (autocrine signaling) as well as in non-infected bystander cells (paracrine signaling). To identify in which cells the transgene activation takes place in context of low IFN levels induced by mCMV, we additionally implemented a P_Tet_-EGFP reporter module in ConvAmp cells (ConvAmp-eGFP) and tracked mIFN-mediated reporter activation in single cells. To this end, ConvAmp-eGFP cells were infected with the mCMV-mCherry reporter virus and subjected to time-lapse microscopy (Figure 4B). Time-resolved image analysis revealed that both, primary infected cells and bystander cells showed upregulation of the ConvAmp-driven EGFP gene, indicating that low amounts of IFN secreted by the virus-infected cells are sufficient to induce the synthetic loop in infected cells as well as uninfected bystander cells. Of note, viral mCherry signals preceded the P_Tet_-EGFP signal in all infected cells that were monitored.

Together the results indicate that the ConvAmp cells can overcome the innate immune block mediated by mCMV antagonistic factors and can convert a low ISG activation into a robust gene expression program. Thus, we have established a boosted device that shows ‘all-or-nothing’ sensitivity in response to viral infections.

### ConvAmp cells convert a transient IFN trigger signal to a sustained antiviral response resulting in efficient protection from viral infection

**Figure 5.**
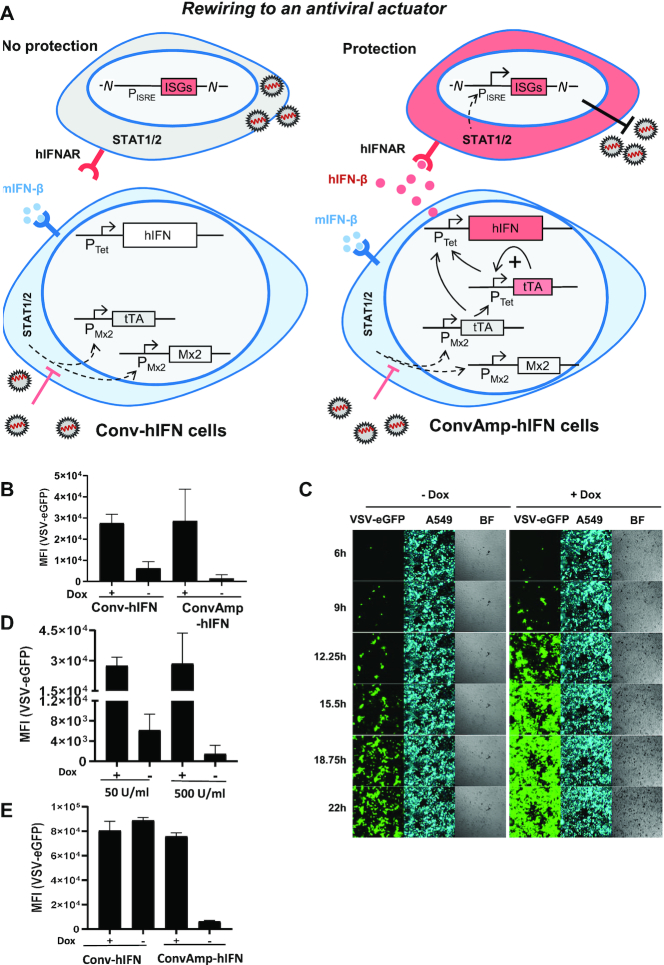
Conversion of the trigger mIFN-β to hIFN-β protects human cells from infection. (**A**) Schematic representation of Conv-hIFN and ConvAmp-hIFN cells and induced protection against viral infection in presence of antagonistic viral proteins. Coloured boxes indicate constantly induced genes, white boxes indicate the situation upon viral counteraction. (**B**) Conv-hIFN and Conv-Amp-hIFN cells were cultured in the absence or presence of doxycycline and stimulated for 6h with 500 U/ml mIFN-β. The supernatant was harvested 24h after treatment. Human A549 cells were pre-incubated for 16 h with the different supernatants and then infected with eGFP tagged VSV for one hour (MOI 1). Viral eGFP expression was analysed 24h post infection by flow cytometry. The experiment was performed once in triplicates. (**C**) ConvAmp-hIFN cells were co-cultured with BFP-tagged A549 cells in a 1:5 ratio in the presence or absence of doxycycline. Cells were stimulated for 6 h with 500 U/ml mIFN-β. 16 h later these cells were infected with VSV (MOI 1) and analysed by time-lapse microscopy. Representative pictures are shown from one experiment performed in triplicates. (**D**) ConvAmp-hIFN cells were co-cultured with BFP-tagged A549 cells in a 1:5 ratio in the presence or absence of doxycycline. Cells were stimulated with 500 U/ml or 50 U/ml mIFN-β for 6 h. 16 h later, these cells were infected with eGFP-tagged VSV (MOI 1). Virus mediated eGFP expression was analysed 24h post infection by flow cytometry. The experiment was performed once in triplicates. (**E**) The Conv-hIFN and ConvAmp-hIFN cells were cultured in the presence or absence of doxycycline. Cells were infected with mCMV (MOI 1). The supernatant was harvested 24 h after infection. A549 cells were pre-incubated with the supernatants for 16 h and then infected with VSV (1 h, MOI1). Viral eGFP expression was analysed 24 h post infection by flow cytometry. The experiment was performed once in triplicates.

We aimed at establishing cells that convert the sensing of infection associated intracellular signals into the production of a robust antiviral activity, thereby generating sensor/actuator cells which convey protection to surrounding cells. Based on the fact that IFNs work highly species specific (36) we employed human IFN-β (hIFN-β) as an actuator that confers antiviral activity in human cells and thus can be discriminated from an initial mIFN-β trigger signal. We transfected ConvAmp or Conv cells with a P_Tet_-hIFN-β cassette to generate ConvAmp-hIFN or Conv-hIFN cells, respectively (Figure 5A).

To evaluate the conversion of the murine trigger (mIFN-β) to the human actuator (hIFN-β), ConvAmp-hIFN and control Conv-hIFN cells were induced with mIFN-β (500 U/ml) for 6h. The supernatants were added to human A549 cells. To test for human specific antiviral activity the A549 cells were infected with an eGFP-tagged vesicular stomatitis virus (VSV). The efficiency of virus infection was assessed by determining the level of eGFP expression in these cells. Pre-incubation of A549 cells with the supernatants of mIFN-β-stimulated ConvAmp-hIFN and Conv-hIFN cells resulted in reduced levels of GFP expression when compared to the levels obtained upon pre-incubation with the supernatants of mIFN-β-stimulated cells in presence of doxycycline. This indicates that hIFN-β produced by ConvAmp-hIFN and Conv-hIFN cells was sufficient to reduce VSV infection. In agreement with the improved expression properties of cells with the amplifier modules, the supernatants from the ConvAmp-hIFN cells provided enhanced protection from VSV infection, indicating that these cells produced higher amounts of hIFN-β (Figure 5B).

To investigate the effect of mIFN-β triggered hIFN-β expression in real time, we co-cultivated mouse ConvAmp-hIFN cells together with human BFP-tagged A549 cells in a ratio of 1:5 and stimulated the co-culture with murine IFN-β. After one day, the cells were infected with VSV-eGFP and subjected to time-lapse microscopy. Kinetic analysis indicates that in the presence of doxycycline, viral eGFP expression was detected in >80% of BFP-positive cells. In contrast, in absence of doxycycline, when the synthetic module was active and hIFN-β could be produced, much less cells were found to be infected over time, indicating that protection of A549 cells was much more efficient (Figure 5C). Even a low concentration of the initial mIFN-β trigger was sufficient for induction of antiviral response in human cells (Supplementary Figure S8) and installed an effective antiviral state in the human A549 target cells as outlined in Figure 5D.

Finally, we investigated if viral infection of ConvAmp-hIFN cells can create protective levels of hIFN-β. ConvAmp-hIFN and Conv-hIFN cells were infected with mCMV. Supernatants of mCMV infected ConvAmp-hIFN cells, but not Conv-hIFN cells induced the antiviral program in human cells which increased with elevated MOIs of mCMV (Supplementary Figure S9). The supernatants were tested for blocking VSV-eGFP infection in human A549 cells. A549 cells pre-treated with the supernatant of mCMV-infected ConvAmp-hIFN cells showed drastically reduced eGFP levels indicating that the human cells could be largely protected from VSV infection. Of note, this protection was abolished when the hIFN-β producer cells were cultivated in presence of doxycycline. In contrast, no decrease in VSV-eGFP infection was observed for A549 cells pretreated with supernatant of Conv-hIFN cells (Figure 5E). Together, this demonstrates that the functional rewiring of infection signals to hIFN-β as a biological actuator protein provides efficient protection from viral infection.

## DISCUSSION

In this study, we exploited the cell-intrinsic type I IFN sense/response cascade for the development of modular sensor/actuator cells that allow visualizing and counteracting viral infection. First, we employed CRISPR/Cas9-assisted targeting of the synthetic transactivator tTA to the IFN-responsive Mx2 promoter to generate a converter module that rewires cell intrinsic IFN sensing to the synthetic, doxycycline dependent transcriptional activator tTA. The Mx2 promoter was chosen because it combines strict IFN-dependent regulation with exclusive induction by IFNs (12). This Conv module represents an IFN-responsive hub which was evaluated with respect to sensitivity and kinetics, using luciferase and EGFP as actuators that convey visualization. Importantly, this revealed the tight control of reporter gene activation upon cellular sensing of mIFN. Thus, the time course of luciferase expression in Conv cells mirrors the transient activity of the Mx2 promoter, which reflects the cell intrinsic negative feedback regulation in the IFN response pathway mediated by factors such as SOCS1 (6). Moreover, mIFN-based activation of luciferase was completely abrogated in presence of doxycycline (Figure 1D). Doxycycline impairs binding of the tTA to its cognate synthetic promoter in a dose dependent manner and thus provides the option to fine-tune and modulate levels of the tTA (37). Together, the Conv module facilitates authentic, immediate and tunable conversion of the physiological trigger (IFN) to synthetic cassettes driving transgene expression.

Despite the fact that Conv cells efficiently sensed cellular IFN production upon NDV infection and monitored the course of IFN induction over time, Conv cells failed to visualize infection with mCMV (Figure 2A). Characterization of Conv cells upon mCMV infection revealed that this was a consequence of mCMV’s ability to largely prevent secretion of mIFN by counteracting its induction (Figure 2C). Several proteins encoded by the mCMV genome counteract both, type I IFN induction downstream of DNA sensors as well as the paracrine or autocrine response towards secreted IFN, which is mediated via the Jak/STAT pathway (38–41). However, various cell types respond differently towards mCMV infection: In contrast to professional IFN producing cells, such as plasmacytoid dendritic cells, which produce high levels of IFN, mCMV infection of fibroblastoid cells results in a weak and short-term induction of IFN activity (Figure 2D and (42)). As a consequence, in mCMV-infected Conv cells the induction of both, the cellular ISG Mx2 as well as the tTA are massively impaired (Figure 2D).

To amplify the low levels of mIFN expression upon infection with mCMV we extended the synthetic circuit and implemented a simple positive feedback module, thereby generating ConvAmp cells. Such modules are characterized by bimodal expression and pronounced hysteresis, i.e. they provide sustained expression even when the trigger is reduced or even absent (35). These positive feedback modules were previously shown to be appropriate for tight control of the cellular proliferation state (43,44), indicating that the expression levels can reach physiologically relevant levels.

The synthetic ConvAmp module developed in this study provides a number of important features that upgrade classical IFN-based reporter systems (8,12). By coupling the endogenous Mx2 promoter activity to the tTA we rewired the signal to a tTA dependent promoter. The incorporation of the P_Tet_-tTA module into the Conv cells created a rapid and efficient system to amplify the IFN-induced transactivator expression, facilitating robust detection of even low amounts of virus-induced IFN (Figure 3E). Such highly sensitive systems could be useful for the detection of low levels of infection in versatile experimental animal models. Moreover, converter systems might be considered for diagnostic purposes, such as the detection of hCMV in transplant recipients to initiate preemptive therapies and prevent the progression of an asymptomatic infection into CMV disease.

In addition to the improved sensitivity, a sustained reporter gene expression is observed in ConvAmp cells upon activation of P_Tet_-tTA (Figure 3D). Single cell analysis revealed that both primary infected cells and bystander cells can sense mIFN-β, indicating that even in presence of the strong antagonistic mechanisms in infected cells the cascade can be induced. Notably, only a fraction of mCMV-infected cells and bystander cells showed expression of EGFP (Figure 4B). This reflects the recent observation that at low IFN concentrations the ISG induction is bimodal in individual cells (9) and thus also Mx2-driven tTA expression is characterized by stochastic activation. Nevertheless, due to the sustained response upon viral infection, these reporter systems should allow identifying cells in which viral infection shuts down IFN gene expression after an initial phase of induction. In this regard, such cells would help to better understand acute viral infections. Moreover, they can be exploited to identify cellular targets of IFN during viral latency and upon reactivation.

Our conclusions concerning the stimulation of the converter modules in infected cells relies on the assumption that autocrine IFN activation induces the Mx2 promoter. However, we cannot exclude that virus infection leads to a direct Mx2 induction through IRF3 activation implying that the converter system may be triggered independently of IFNAR signaling. Based on our data we formally cannot distinguish between both alternatives, although a number of earlier reports exclude a direct stimulation of Mx2 by virus infection (45,46). But even in case of an IFN-independent stimulation the amplified signal would results in exactly the same out-put as through IFN signaling.

We implemented the Mx-tTA module into mouse embryonic stem cells, which represent a model for human stem cells such as induced pluripotent stem cells. Stem cells offer the opportunity to generate various cell types of interest upon differentiation in vitro. Moreover, they show higher susceptibility towards targeted genetic modifications, thereby providing immediate access to genetically modified primary cells of various tissues.

The instrumentalization of viral infections with the aim to induce therapeutic countermeasures represents a major area of current biomedical research. However, overexpression of a therapeutic gene product with a narrow therapeutic window may be toxic and has to be tightly controlled. Thus, a strictly controlled system is needed for such applications. So far, sensing infection and induction of counteracting strategies are two separate processes during treatment. In the era of synthetic biology, however, it is tempting to envisage cell based systems in which sensing and counteraction are combined and expression can be controlled. Such theranostic sensor/actuator systems could be transplanted into patients at an early stage of infection. In this regard, they would give the chance to monitor the course of infection and induce treatment on demand, i.e. according to the amount of virus generated. Encapsulation of cells would ensure protection from host's immune reactions and potential surgical removal (47), which would meet additional safety criteria of cell therapeutic strategies.

The modules developed in this study could be beneficial for future concepts to treat various diseases. In patients chronically infected with viruses such as hCMV or Hepatitis C Virus (HCV), such theranostic cells could indicate viral bursts and immediately initiate antiviral counteraction, even before clinical symptoms are visible. In a longer perspective, such cell implants might also be envisaged as a prophylactic measure that could protect patients from de novo infections. ConvAmp modules might also be of interest to treat autoimmune diseases that are characterized by dysregulated type I IFN responses such as lupus erythematosus (LE). In such context, flares of LE, which are associated with increased IFN levels, could be monitored and counteracted.

By using hIFN-β as an example of actuator proteins we verify that immediate detection of viral infection leads to the robust and controllable rewiring to therapeutic targets. Besides hIFN-β and other type I IFN family members in particular type III IFNs represent attractive candidates due to the induction of antiviral genes in epithelial cells while largely avoiding deleterious immunomodulatory effects (48). Also, more specific therapeutic targets such as neutralizing antibodies that impair virus spread, the induced secretion of small regulatory RNAs via exosomes can be foreseen. Similarly, the induction of cytokines that improve tissue regeneration in the course of infection or dampen overshooting cytokine storms (e.g. IL10) might be considered. Finally, the combined release of various factors can be envisaged, either by including additional modules in the sensor/actor cells or by combining different cells in the grafts. The presented system could easily be modified for other purposes. The sensor could be replaced by other receptors like PRRs and combined with appropriate natural or synthetic converter modules which become connected to the amplification tool introduced in this work.

So far, this interfaced and tightly controlled sensor/actuator system provides proof of concept for rewiring infection associated responses to actuators of choice that might be of interest for further developing this concept for therapeutic purposes. In this regard, various antiviral actuators should be considered that counteract infections on various levels. Besides the cellular antiviral actuators such as type I IFN which was used in this study as a model actuator, also the induction of other (combinations of) antiviral factors would be of interest. Together, such a development could eventually facilitate efficient sensing and counteracting viral infections for therapeutic purposes. Moreover, given the fact that IFN is also released in response to non-viral infections and even activated in sterile inflammatory conditions the toolbox provided here might be also of interest for other disease states including tumor development.

## Supplementary Material

gkaa961_Supplemental_File

## Notes

Present address: Jan Riedel, Department of Gastrointestinal Oncology, University Hospital, 06097 Halle, Germany.
