# Supplementary material for: Synthetic rewiring and boosting type I interferon responses for visualization and counteracting viral infections
Source: Nucleic Acids Res. 2020 Nov 2;48(20):11799–811. doi: 10.1093/nar/gkaa961 (PMC7672444; doi:10.1093/nar/gkaa961)
Supplement: gkaa961_Supplemental_File [file gkaa961_supplemental_file.pptx]

## Slide 1
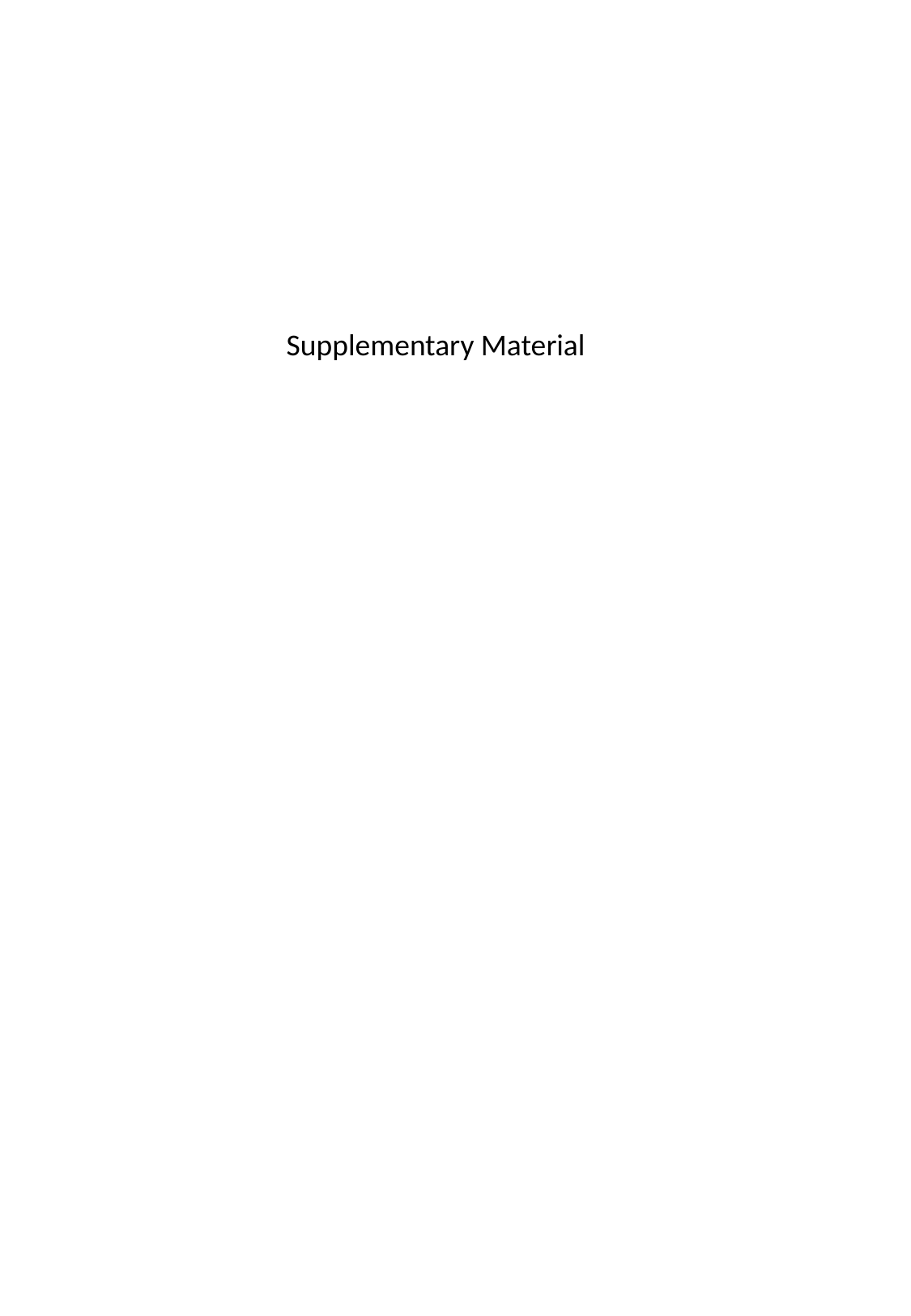

Supplementary Material

## Slide 2
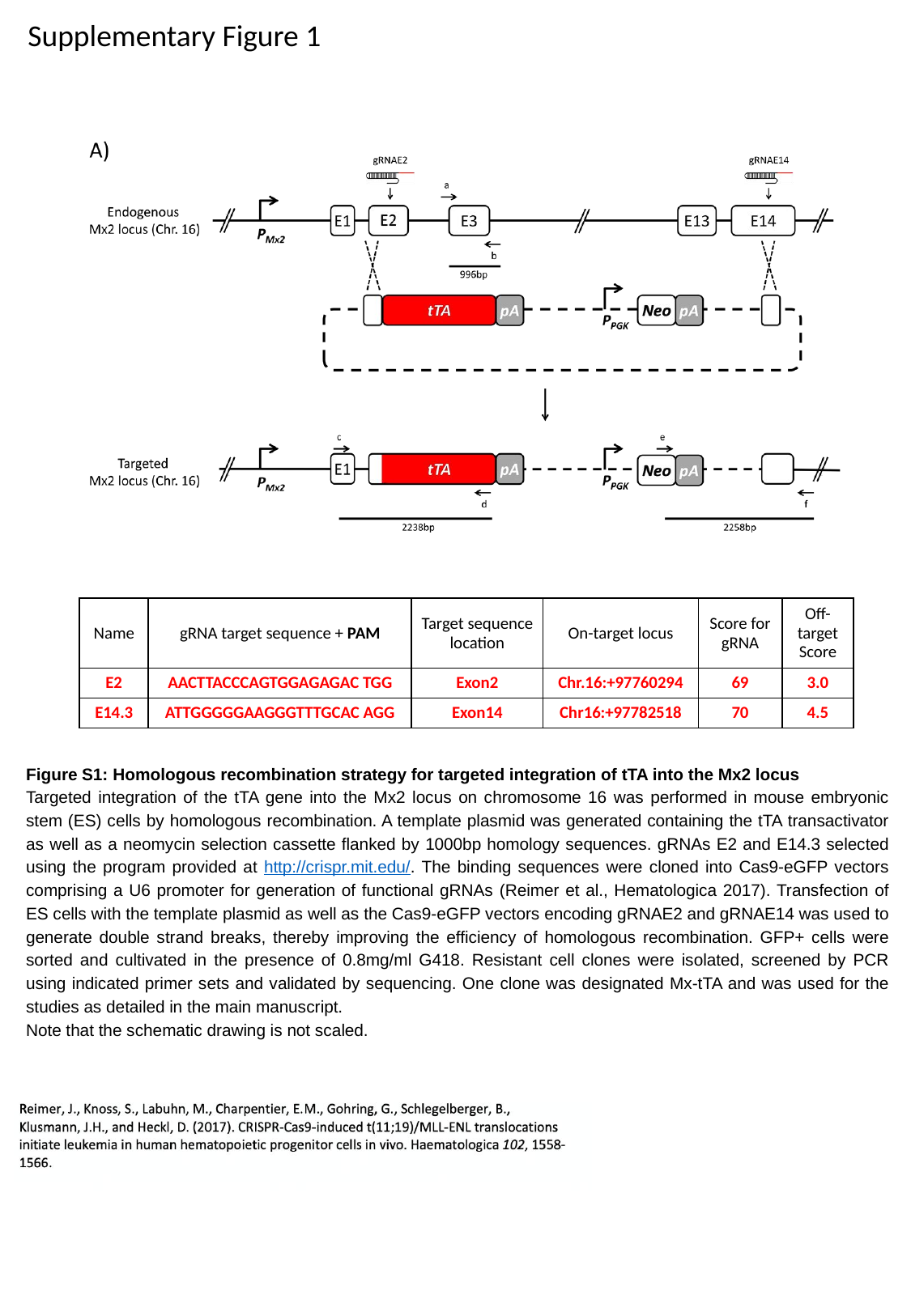

Supplementary Figure 1
| Name | gRNA target sequence + PAM | Target sequence location | On-target locus | Score for gRNA | Off-target Score |
| --- | --- | --- | --- | --- | --- |
| E2 | AACTTACCCAGTGGAGAGAC TGG | Exon2 | Chr.16:+97760294 | 69 | 3.0 |
| E14.3 | ATTGGGGGAAGGGTTTGCAC AGG | Exon14 | Chr16:+97782518 | 70 | 4.5 |
Figure S1: Homologous recombination strategy for targeted integration of tTA into the Mx2 locus
Targeted integration of the tTA gene into the Mx2 locus on chromosome 16 was performed in mouse embryonic stem (ES) cells by homologous recombination. A template plasmid was generated containing the tTA transactivator as well as a neomycin selection cassette flanked by 1000bp homology sequences. gRNAs E2 and E14.3 selected using the program provided at http://crispr.mit.edu/. The binding sequences were cloned into Cas9-eGFP vectors comprising a U6 promoter for generation of functional gRNAs (Reimer et al., Hematologica 2017). Transfection of ES cells with the template plasmid as well as the Cas9-eGFP vectors encoding gRNAE2 and gRNAE14 was used to generate double strand breaks, thereby improving the efficiency of homologous recombination. GFP+ cells were sorted and cultivated in the presence of 0.8mg/ml G418. Resistant cell clones were isolated, screened by PCR using indicated primer sets and validated by sequencing. One clone was designated Mx-tTA and was used for the studies as detailed in the main manuscript.
Note that the schematic drawing is not scaled.

## Slide 3
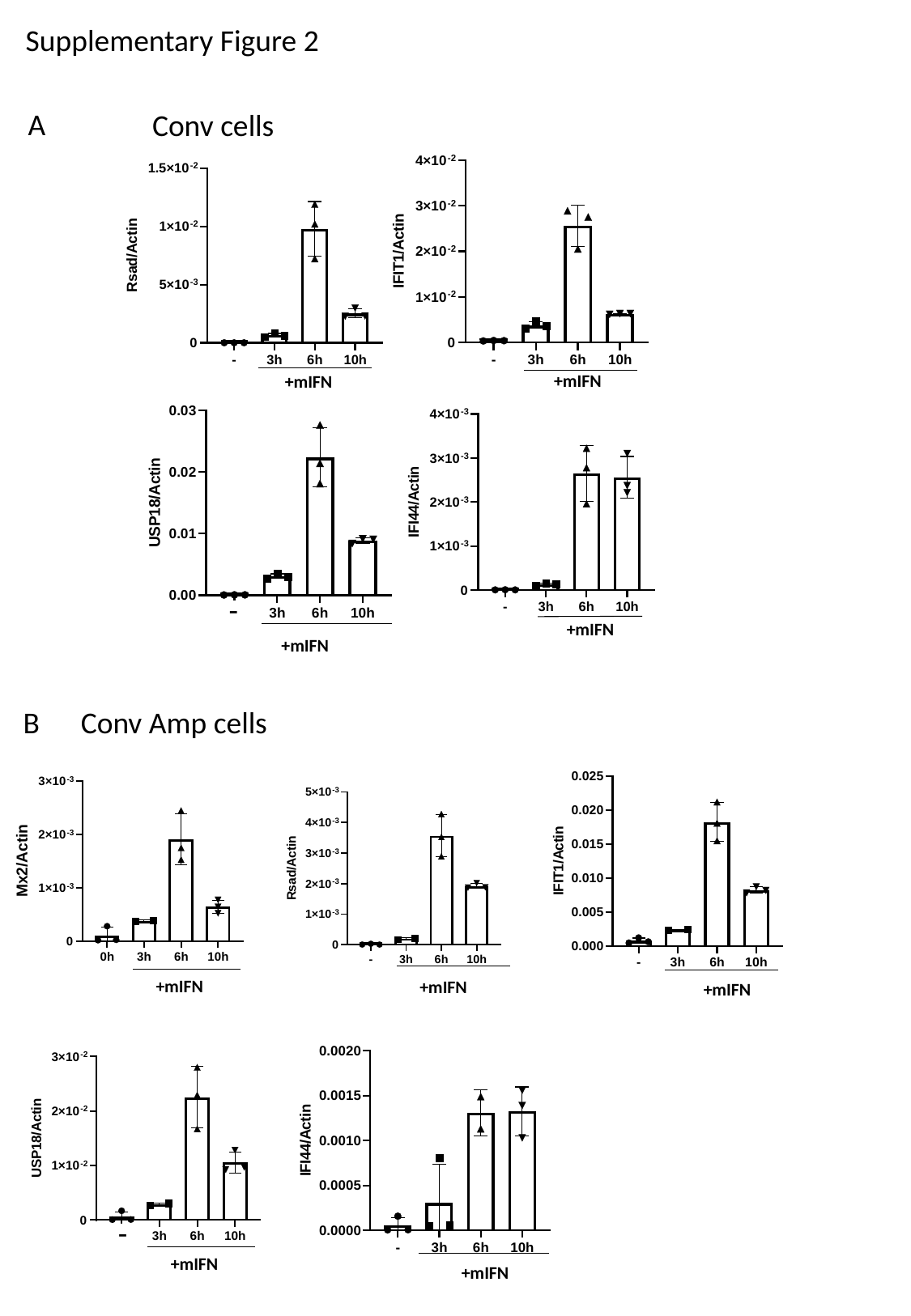

Supplementary Figure 2
A
Conv cells
+mIFN
+mIFN
-
+mIFN
+mIFN
B Conv Amp cells
+mIFN
+mIFN
+mIFN
-
+mIFN
+mIFN

## Slide 4
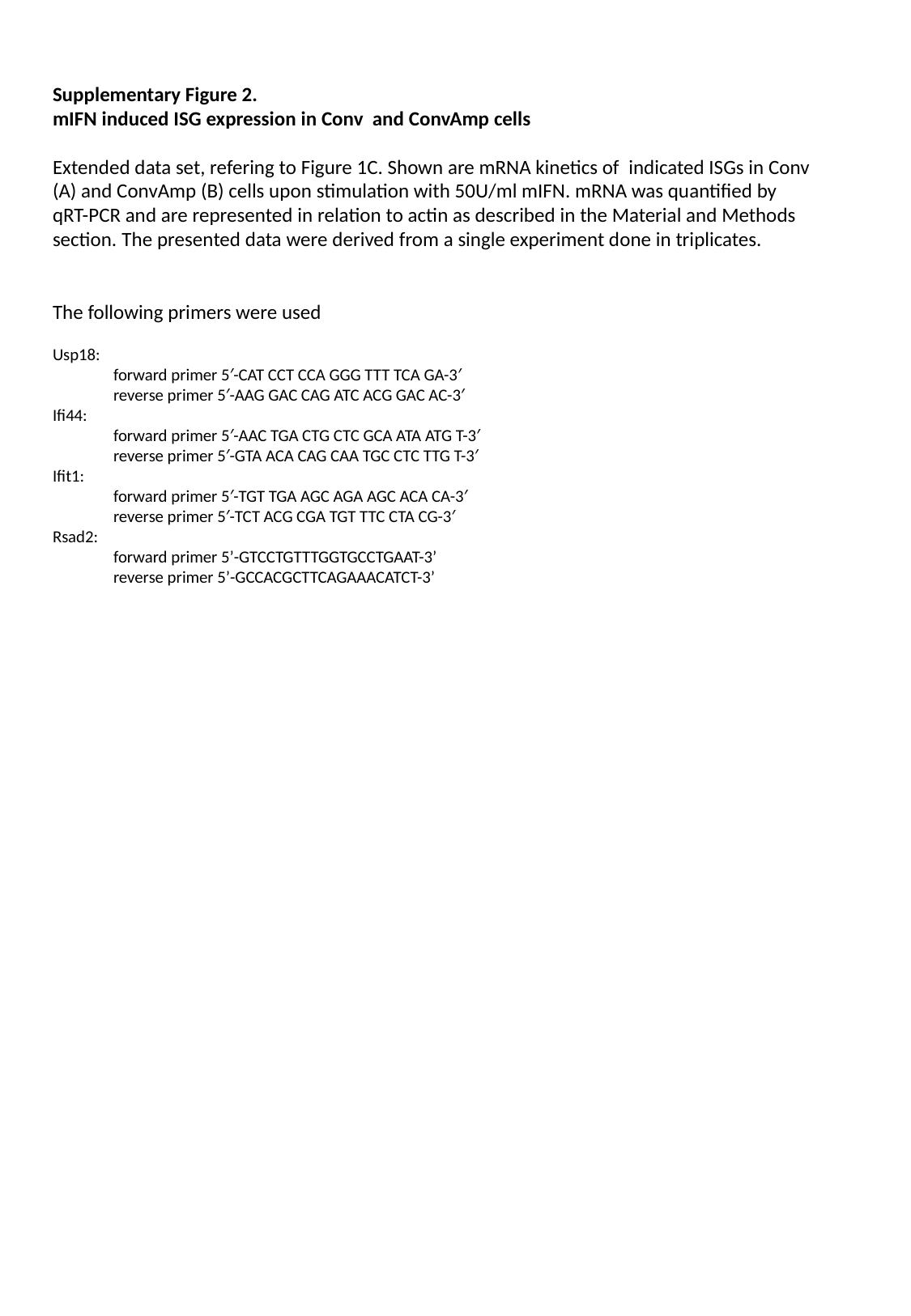

Supplementary Figure 2.
mIFN induced ISG expression in Conv and ConvAmp cells
Extended data set, refering to Figure 1C. Shown are mRNA kinetics of indicated ISGs in Conv (A) and ConvAmp (B) cells upon stimulation with 50U/ml mIFN. mRNA was quantified by qRT-PCR and are represented in relation to actin as described in the Material and Methods section. The presented data were derived from a single experiment done in triplicates.
The following primers were used
Usp18:
forward primer 5′-CAT CCT CCA GGG TTT TCA GA-3′
reverse primer 5′-AAG GAC CAG ATC ACG GAC AC-3′
Ifi44:
forward primer 5′-AAC TGA CTG CTC GCA ATA ATG T-3′
reverse primer 5′-GTA ACA CAG CAA TGC CTC TTG T-3′
Ifit1:
forward primer 5′-TGT TGA AGC AGA AGC ACA CA-3′
reverse primer 5′-TCT ACG CGA TGT TTC CTA CG-3′
Rsad2:
forward primer 5’-GTCCTGTTTGGTGCCTGAAT-3’
reverse primer 5’-GCCACGCTTCAGAAACATCT-3’

## Slide 5
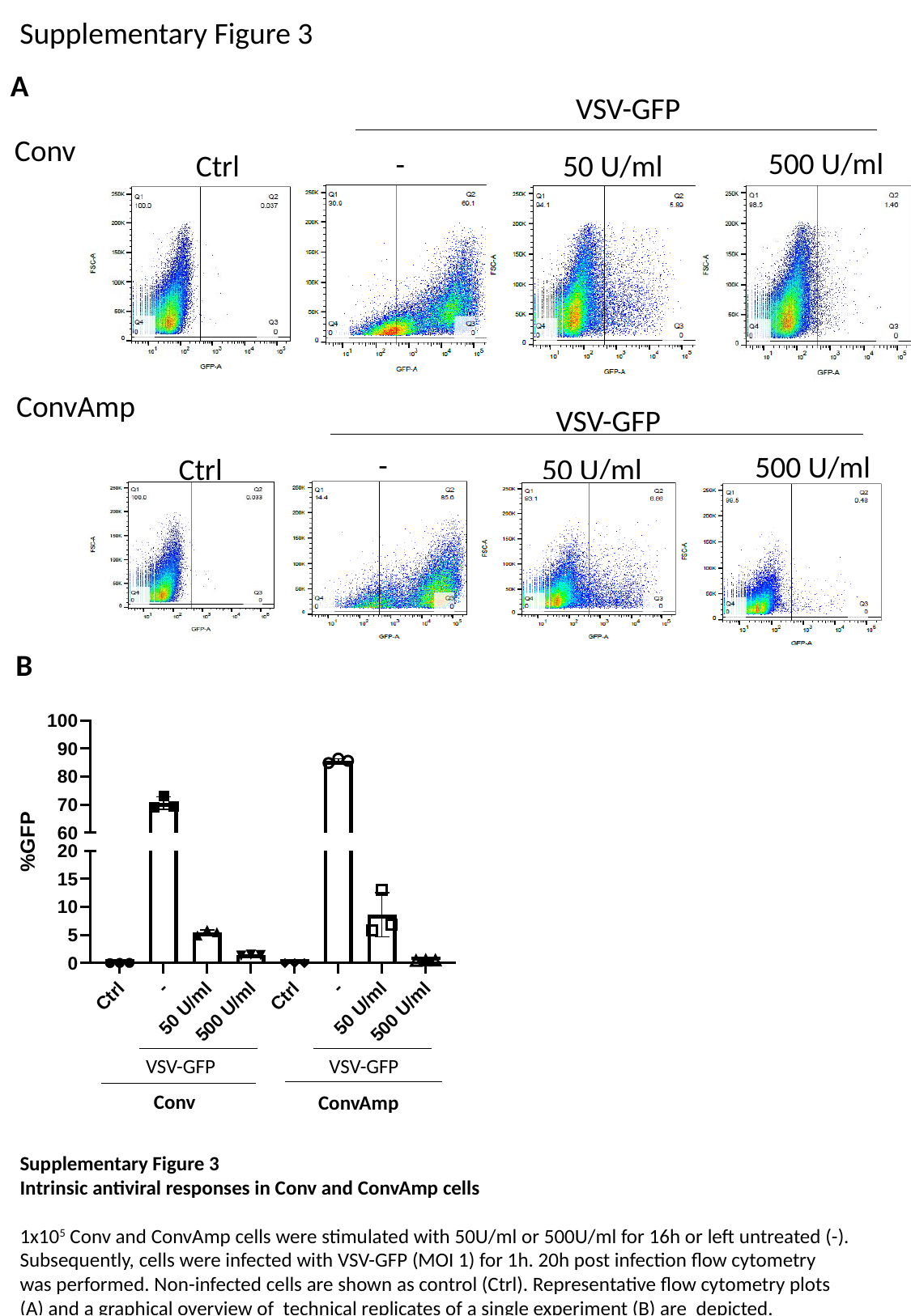

Supplementary Figure 3
A
VSV-GFP
Conv
500 U/ml
-
Ctrl
50 U/ml
ConvAmp
VSV-GFP
-
500 U/ml
Ctrl
50 U/ml
B
VSV-GFP
VSV-GFP
Conv
ConvAmp
Supplementary Figure 3
Intrinsic antiviral responses in Conv and ConvAmp cells
1x10^5^ Conv and ConvAmp cells were stimulated with 50U/ml or 500U/ml for 16h or left untreated (-). Subsequently, cells were infected with VSV-GFP (MOI 1) for 1h. 20h post infection flow cytometry was performed. Non-infected cells are shown as control (Ctrl). Representative flow cytometry plots (A) and a graphical overview of technical replicates of a single experiment (B) are depicted.

## Slide 6
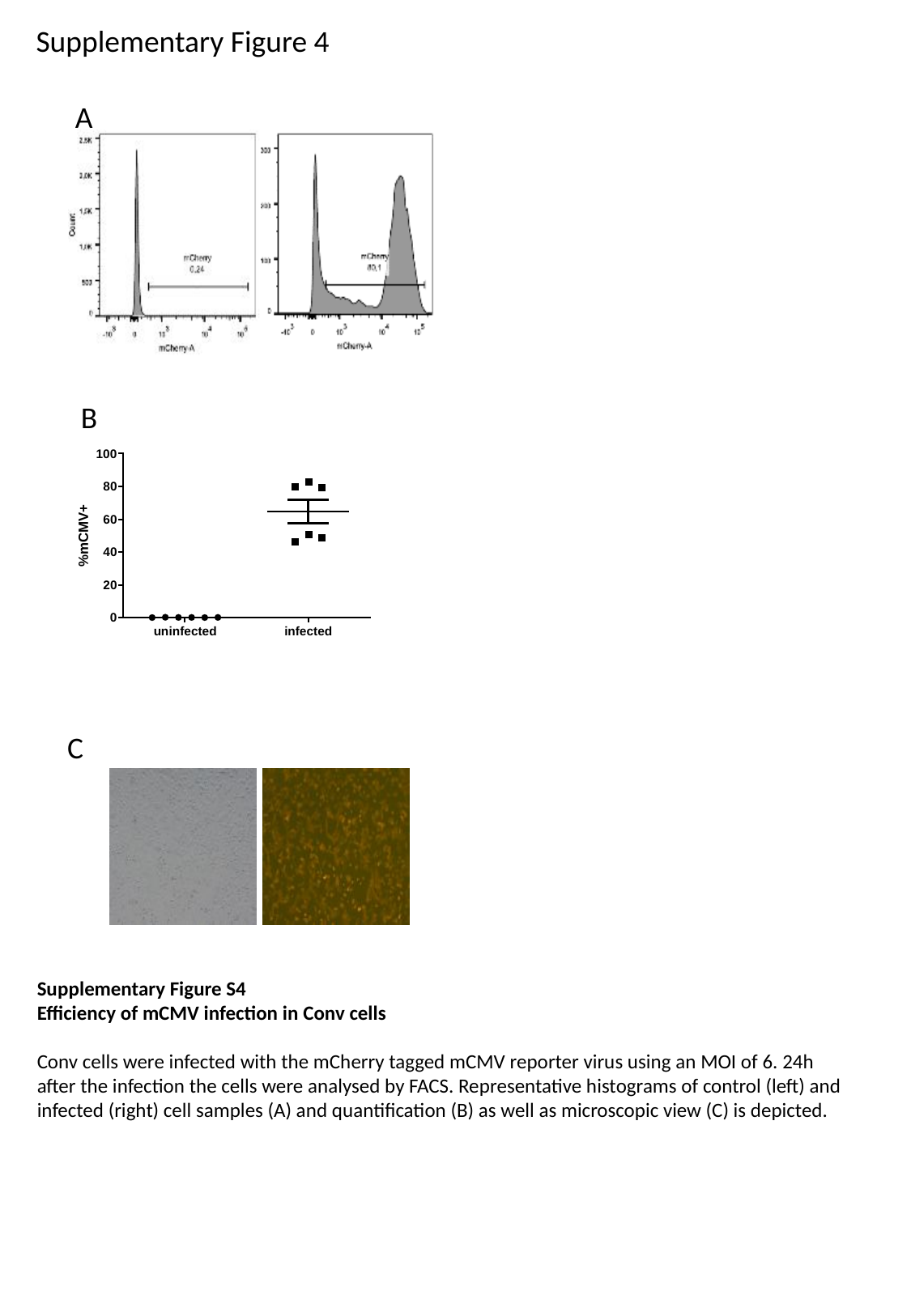

Supplementary Figure 4
A
B
C
Supplementary Figure S4
Efficiency of mCMV infection in Conv cells
Conv cells were infected with the mCherry tagged mCMV reporter virus using an MOI of 6. 24h after the infection the cells were analysed by FACS. Representative histograms of control (left) and infected (right) cell samples (A) and quantification (B) as well as microscopic view (C) is depicted.

## Slide 7
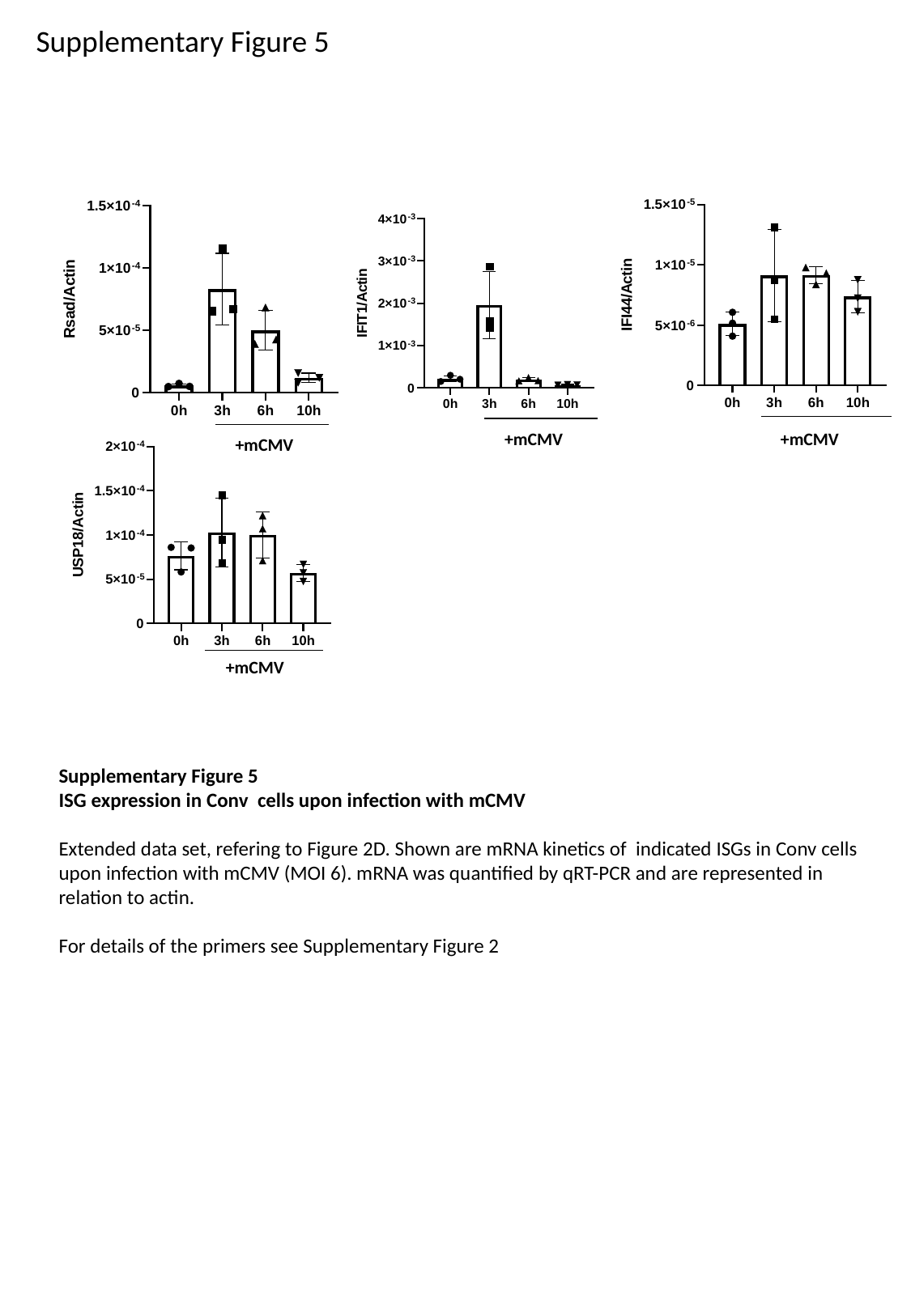

Supplementary Figure 5
+mCMV
+mCMV
+mCMV
+mCMV
Supplementary Figure 5
ISG expression in Conv cells upon infection with mCMV
Extended data set, refering to Figure 2D. Shown are mRNA kinetics of indicated ISGs in Conv cells upon infection with mCMV (MOI 6). mRNA was quantified by qRT-PCR and are represented in relation to actin.
For details of the primers see Supplementary Figure 2

## Slide 8
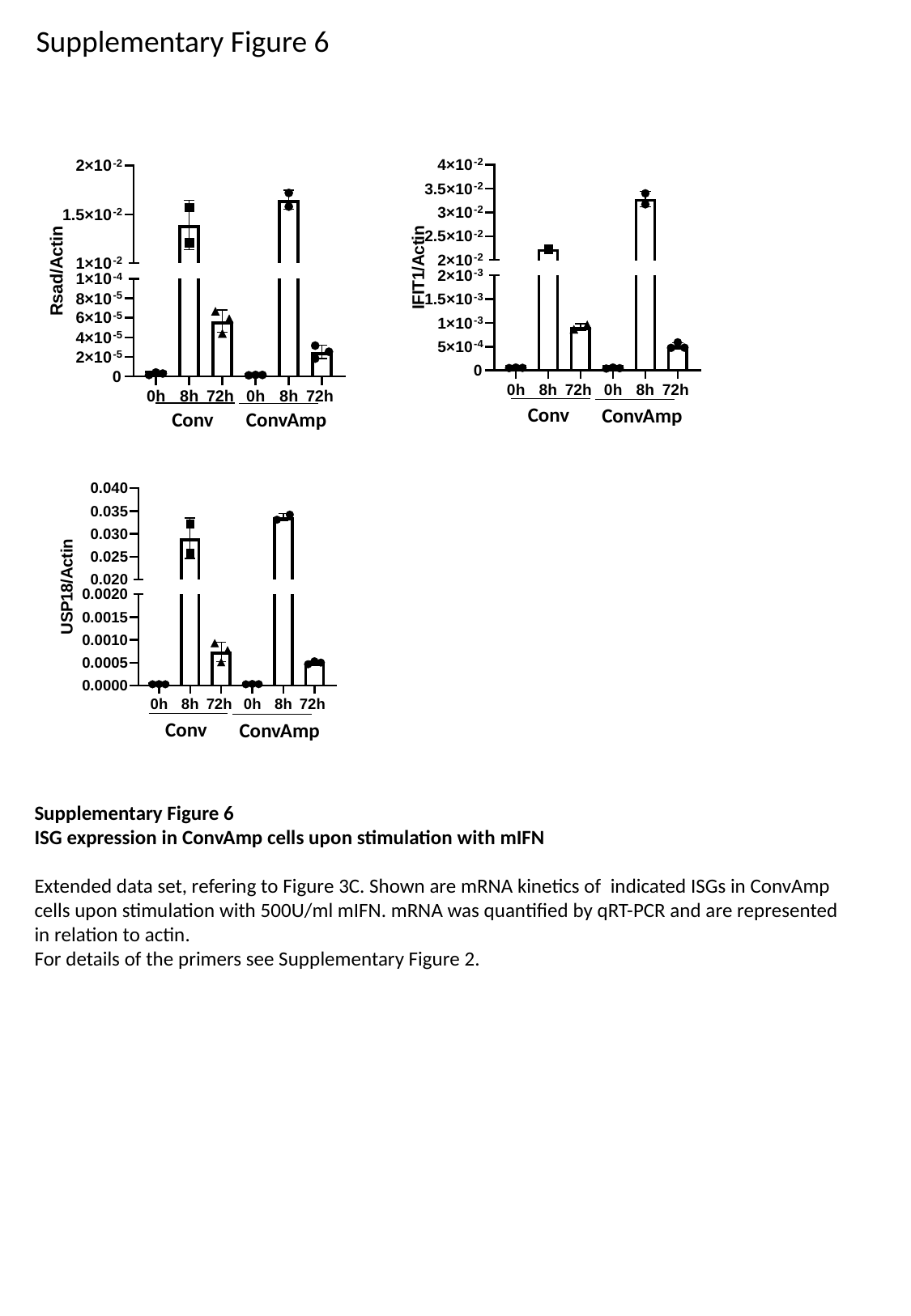

Supplementary Figure 6
Conv
ConvAmp
Conv
ConvAmp
Conv
ConvAmp
Supplementary Figure 6
ISG expression in ConvAmp cells upon stimulation with mIFN
Extended data set, refering to Figure 3C. Shown are mRNA kinetics of indicated ISGs in ConvAmp cells upon stimulation with 500U/ml mIFN. mRNA was quantified by qRT-PCR and are represented in relation to actin.
For details of the primers see Supplementary Figure 2.

## Slide 9
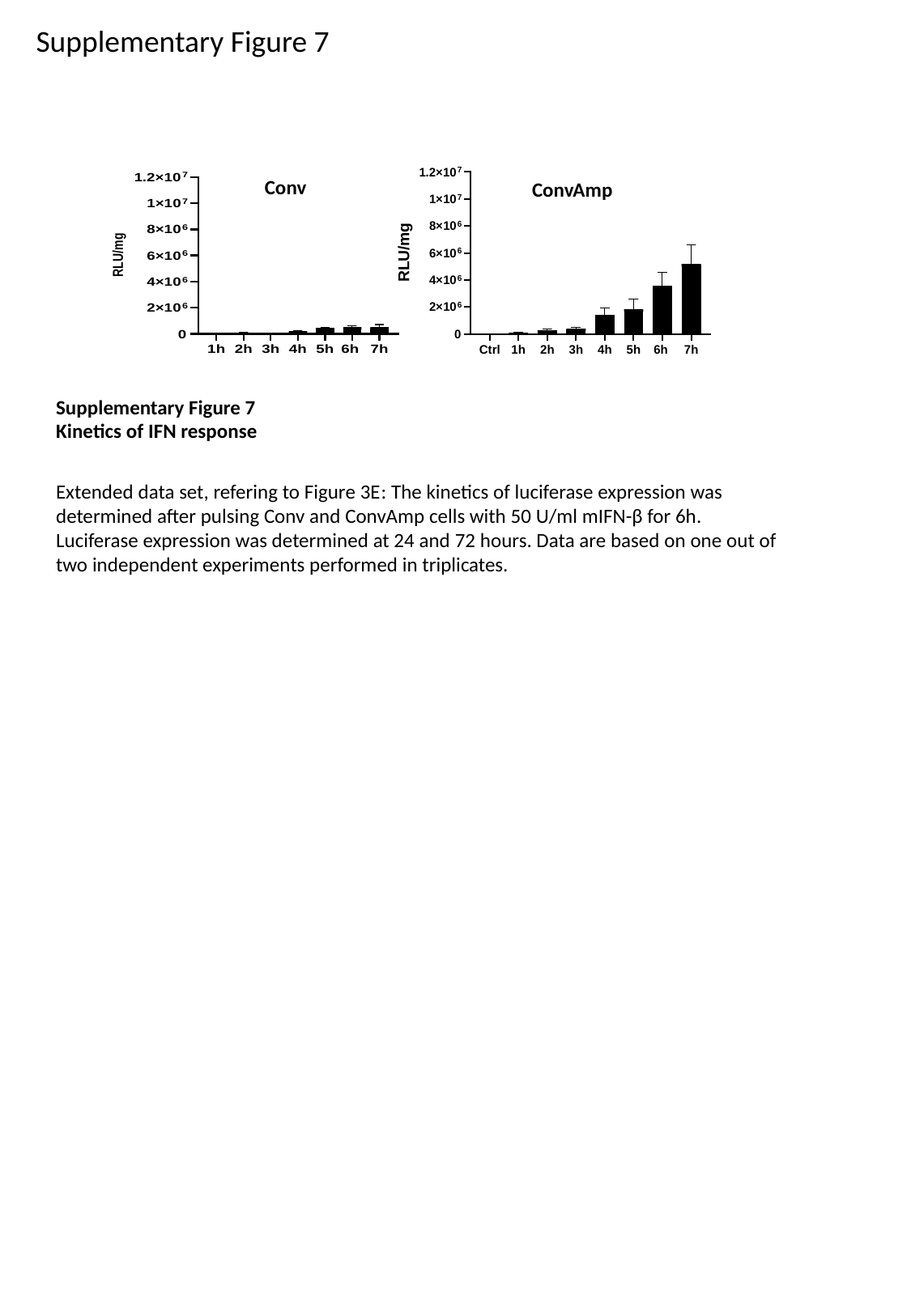

Supplementary Figure 7
Conv
ConvAmp
Supplementary Figure 7
Kinetics of IFN response
Extended data set, refering to Figure 3E: The kinetics of luciferase expression was determined after pulsing Conv and ConvAmp cells with 50 U/ml mIFN-β for 6h. Luciferase expression was determined at 24 and 72 hours. Data are based on one out of two independent experiments performed in triplicates.

## Slide 10
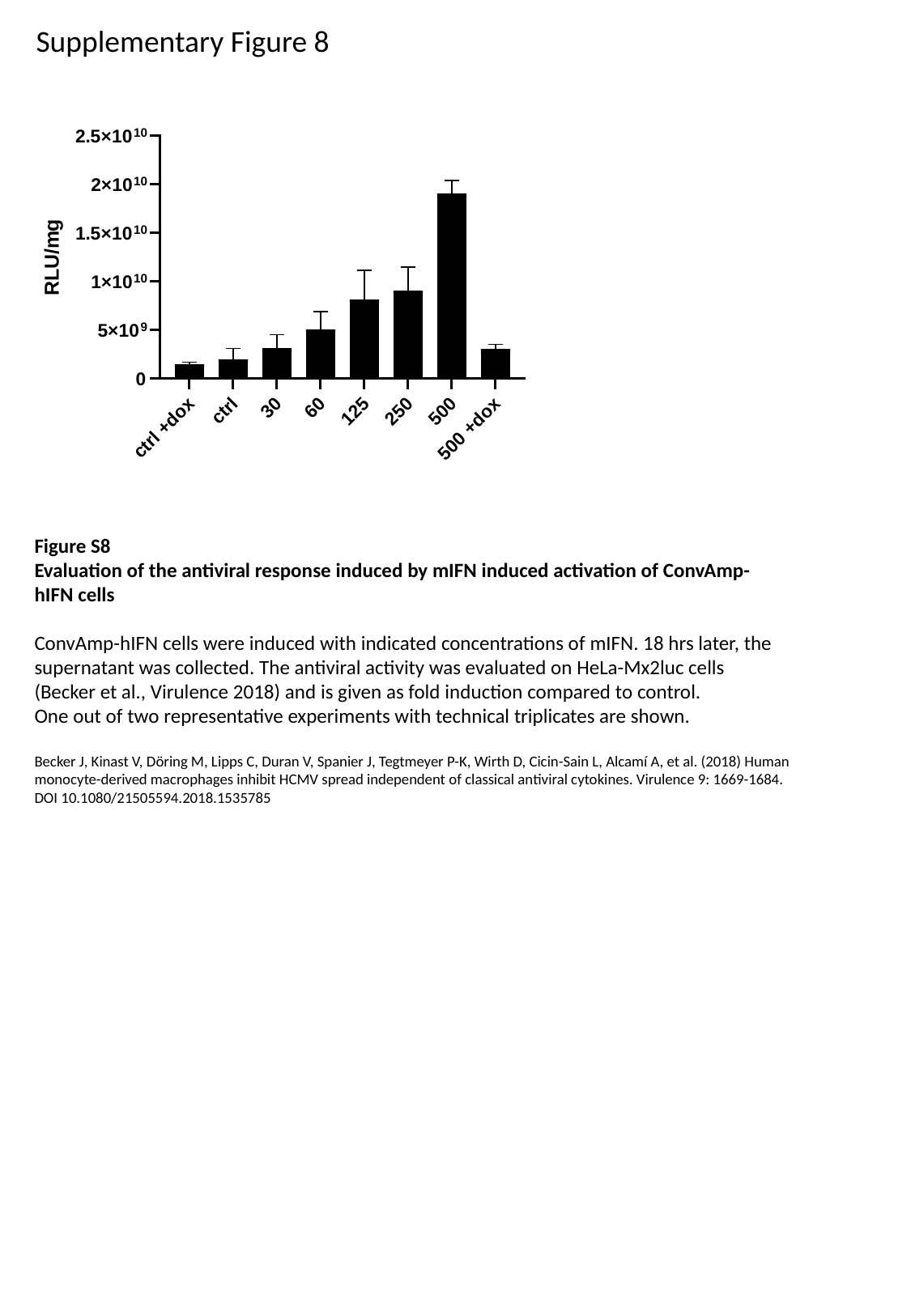

Supplementary Figure 8
Figure S8
Evaluation of the antiviral response induced by mIFN induced activation of ConvAmp-hIFN cells
ConvAmp-hIFN cells were induced with indicated concentrations of mIFN. 18 hrs later, the supernatant was collected. The antiviral activity was evaluated on HeLa-Mx2luc cells (Becker et al., Virulence 2018) and is given as fold induction compared to control.
One out of two representative experiments with technical triplicates are shown.
Becker J, Kinast V, Döring M, Lipps C, Duran V, Spanier J, Tegtmeyer P-K, Wirth D, Cicin-Sain L, Alcamí A, et al. (2018) Human monocyte-derived macrophages inhibit HCMV spread independent of classical antiviral cytokines. Virulence 9: 1669-1684. DOI 10.1080/21505594.2018.1535785

## Slide 11
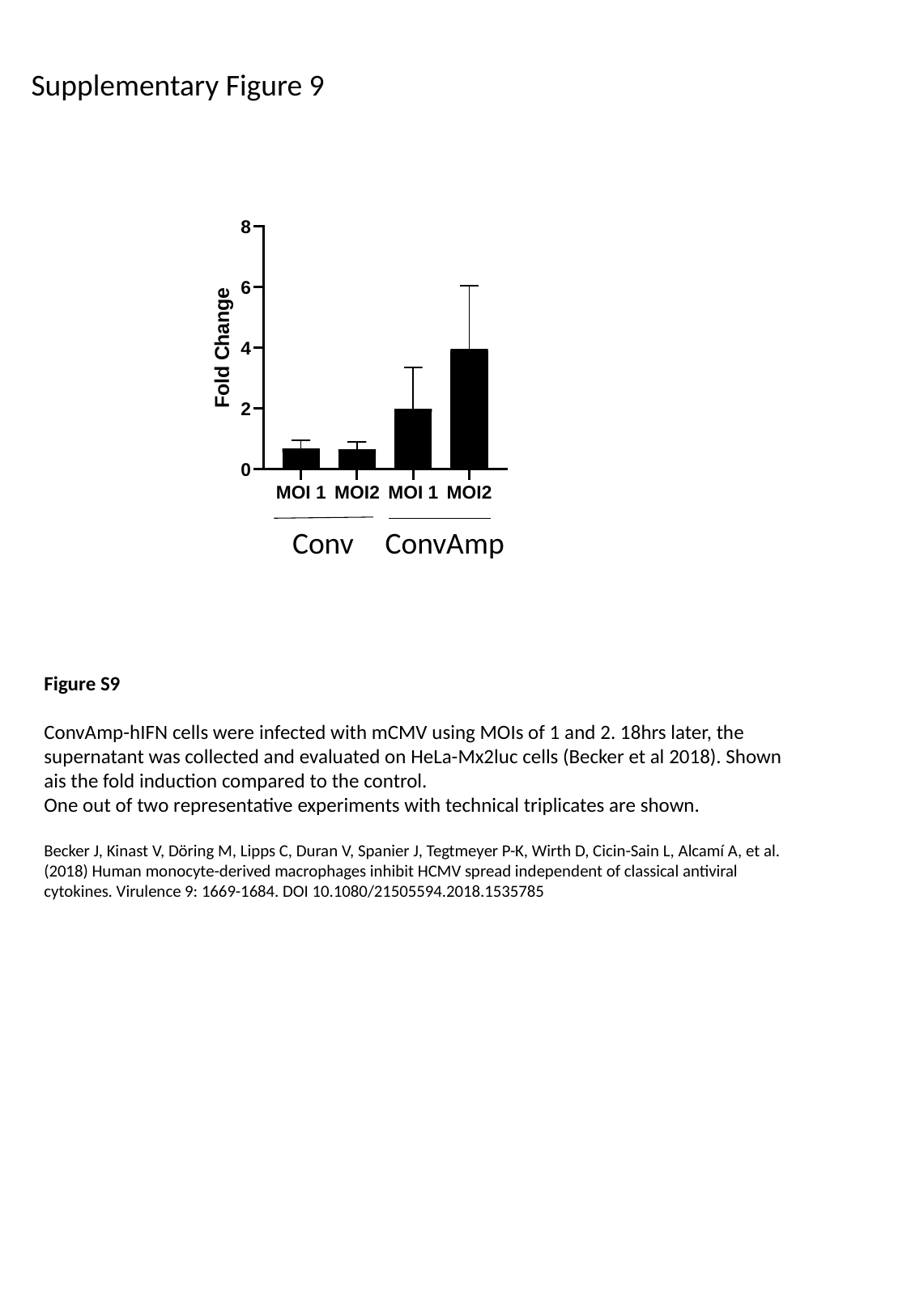

Supplementary Figure 9
Conv
ConvAmp
Figure S9
ConvAmp-hIFN cells were infected with mCMV using MOIs of 1 and 2. 18hrs later, the supernatant was collected and evaluated on HeLa-Mx2luc cells (Becker et al 2018). Shown ais the fold induction compared to the control.
One out of two representative experiments with technical triplicates are shown.
Becker J, Kinast V, Döring M, Lipps C, Duran V, Spanier J, Tegtmeyer P-K, Wirth D, Cicin-Sain L, Alcamí A, et al. (2018) Human monocyte-derived macrophages inhibit HCMV spread independent of classical antiviral cytokines. Virulence 9: 1669-1684. DOI 10.1080/21505594.2018.1535785

## Slide 12
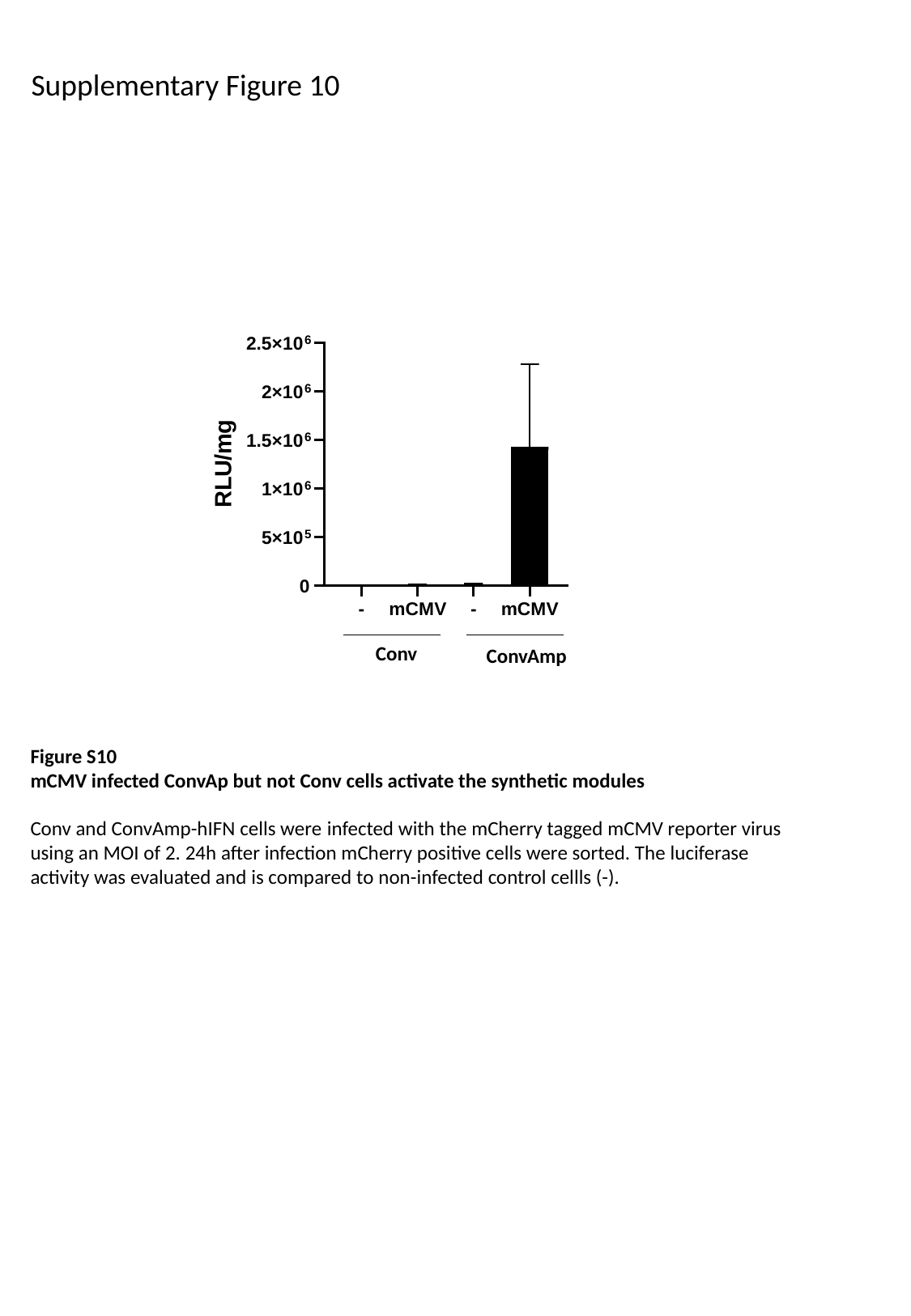

Supplementary Figure 10
Conv
ConvAmp
Figure S10
mCMV infected ConvAp but not Conv cells activate the synthetic modules
Conv and ConvAmp-hIFN cells were infected with the mCherry tagged mCMV reporter virus using an MOI of 2. 24h after infection mCherry positive cells were sorted. The luciferase activity was evaluated and is compared to non-infected control cellls (-).
